# The Impact of Prophylactic Polishing Pastes on the Surface Roughness, Color, and Stain Resistance of CAD/CAM Restorative Materials

**DOI:** 10.3390/dj13050212

**Published:** 2025-05-15

**Authors:** Betul Arkan, Ipek Iscan, Neslihan Tinastepe

**Affiliations:** 1 Department of Prosthodontics, Institute of Health Sciences, Faculty of Dentistry, Istanbul Medipol University, Istanbul 34250, Turkey; betularkann@gmail.com; 2 Department of Prosthodontics, Faculty of Dentistry, Istanbul Medipol University, Istanbul 34214, Turkey; iiscan@medipol.edu.tr; 3 Department of Prosthodontics, Faculty of Dentistry, Uskudar University, Istanbul 34768, Turkey

**Keywords:** prophylactic polishing paste, CAD/CAM, surface roughness, discoloration

## Abstract

**Background:** Prophylactic polishing pastes (PPPs) are widely used to clean teeth and dental restorations; however, their effects on restorative materials are crucial for clinical outcomes. This study investigates the impact of PPPs on the susceptibility of CAD/CAM restorative materials to staining and the relationship between surface roughness and discoloration. **Methods:** Samples of tested materials (resin nanoceramic, hybrid ceramic, feldspathic ceramic, and lithium disilicate-reinforced glass ceramic) were treated with various PPPs (Cleanic, CleanJoy, Detartrine, Proxyt). Surface roughness and color parameters were recorded before and after the PPP application and following coffee immersion for 12 days. Initial measurements of surface roughness (Ra1) and color were taken. The specimens were divided into groups based on the PPP applied. After PPP application, secondary roughness (Ra2) and color values were measured. Changes in roughness (ΔRa), color (ΔE*ab, ΔE00), and whiteness index (ΔWID) were calculated post-application and after coffee immersion. Data normality was tested with the Shapiro–Wilks test. Two-way ANOVA evaluated the effects of material type and PPP on ΔE, ΔWID, and ΔRa. One-way ANOVA, Tukey HDS test, and Pearson correlation were used for further analysis, with significance set at *p* < 0.05. **Results:** The Vita Enamic–Detartrine group showed the highest ΔEab 1 and ΔE001 values, while Cerasmart–control showed the lowest. The Vita Enamic–Proxyt group had the lowest ΔWID1, and Vita Enamic–Cleanjoy exhibited the highest values of ΔEab 2, ΔE002, and ΔWID2. The E.MAX–control group had the lowest values for these metrics. No significant correlation was found between ΔRa and color changes (ΔE*ab 2, ΔE002, ΔWID2); however, a moderate positive correlation was found between values of ΔE1 and ΔE2. **Conclusions:** These findings indicate that PPPs significantly affect the discoloration and surface features of CAD/CAM materials, with both types of PPPs and materials.

## 1. Introduction

In recent years, significant advancements have emerged in CAD/CAM (Computer-Aided Design/Computer-Aided Manufacture) technology within modern dentistry. Despite the well-established effectiveness of CAD/CAM materials in achieving precise color matching and ensuring high quality [1,2,3], various factors contribute to the discoloration of both resin-based and ceramic CAD/CAM materials within the oral environment, stemming from intrinsic and extrinsic factors [4,5].

Patients undergoing prosthodontic treatment, similar to other individuals, maintain regular visits to the dentist for professional teeth cleaning at defined intervals. Within this protocol, clinicians frequently employ prophylactic polishing pastes (PPPs) to cleanse both natural teeth and restorations [6]. Various types of pastes with different compositions are available in the market and are capable of being applied in either single or multiple steps to achieve the required cleaning and smoothing on various surfaces [7,8]

PPP application involves the use of specially designed rubber cups or brushes. Similar to toothpaste, PPPs typically contain binders, humectants, coloring agents, preservatives, fluoride, flavorings, and abrasives in coarser finishes [9].

Drawbacks of prophylactic pastes include the removal of the fluoride-rich enamel layer, and the potential creation of scratches and matting on restorative materials, leading to color changes and increased surface roughness, ultimately causing greater plaque accumulation [7,8,9,10,11]. Elevated biofilm accumulation, indicated when roughness reaches a clinically acceptable threshold of 0.2 μm, triggers gingival inflammation and facilitates secondary caries, compromising the lifespan of restorations [4,10].

Color stability and consistency are crucial clinical factors in esthetic dental restoration. Color differences (ΔE) can be identified either by the naked eye or with the assistance of instruments. For the analysis of dental material color characteristics, spectrophotometers are employed, determining color coordinates such as L value (lightness/darkness), a* value (red-green chromaticity), and b* value (yellow-blue chromaticity). The widely used CIELAB color difference formula (ΔE*ab) aids dental research in this context [4,12].

The CIEDE 2000 color system, introduced by CIE in 2000 alongside the development of the CIELAB color system, is prevalent in dental studies due to increased color parameters and a better-adapted assessment of color difference compared to the CIELAB formula. Recent dental research indicates its effectiveness in assessing color differences between tooth colors [12]. Both formulas were employed in this study. Moreover, a recently introduced whiteness index referred to as WID and grounded in the CIELAB color space was employed to assess the efficacy of diverse therapeutic approaches within the realm of dental practices [13].

Threshold values for perceptible and acceptable color changes in the CIELAB system are acknowledged as ΔE*ab > 1.2 and ΔE*ab ≤ 2.7, respectively, within the dental professional community. Similarly, for the CIEDE 2000 system, these values are established as ΔE00 > 0.8 and ΔE00 ≤ 1.8. The corresponding thresholds for ΔWID are considered to be 0.72 and 2.60 [4,14].

Overall, prophylactic polishing pastes may induce surface roughness in CAD/CAM restorative materials, heightening their susceptibility to staining when exposed to staining solutions. Although various studies have reported adverse effects of PPP on natural teeth surfaces [15,16] and CAD/CAM materials [17,18], a comparative analysis of the diverse effects of polishing protocols on surface properties with CAD/CAM resin materials [6,19] is lacking. The evaluation of PPPs’ effect on the stainability of CAD/CAM restorative materials after immersion in a staining solution and the correlation of surface roughness with discoloration using the CIELAB (ΔE*ab), CIEDE 2000 (ΔE00), and Whiteness change (ΔWID) constitute the primary objectives. The null hypotheses of this in-vitro study are defined as follows: “Prophylactic pastes have no effect on the stainability of CAD/CAM resin restorative materials, and there is no correlation between surface roughness change after PPP application and discoloration after immersion in a coffee solution”.

## 2. Material and Methods

**Figure 1:**
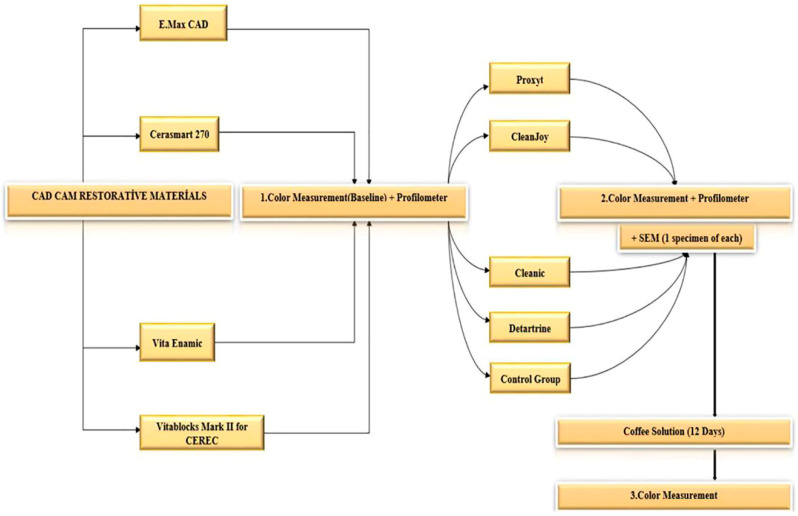
A diagram of the study design.

The CAD/CAM blocks encompassed a feldspathic ceramic (Vitablocs Mark II), a hybrid ceramic block (Vita Enamic), a resin nanoceramic (Cerasmart), and a lithium disilicate glass ceramic (E.MAX-CAD) (Table 1). A schematic representation of the study’s design is depicted in Figure 1. The CAD/CAM blocks were sectioned using a water-cooled, low-speed diamond saw (Isomet, Buehler, IL, USA). Thirty samples were generated, each material having a thickness of 1 mm. Among these samples, five were specifically allocated for scanning electron microscopy (SEM) assessment. The E.MAX-CAD specimens underwent a crystallization process in a ceramic furnace, following the manufacturer’s instructions. Both surfaces of all samples underwent polishing (Phoenix Beta, Buechler, IL, USA) using abrasive silicon carbide papers (English Abrasives & Chemicals Ltd., London, UK) with decreasing grain sizes (600, 800, 1000, and 1200 grit) under water-cooling for 60 s. Subsequently, the specimens were ultrasonically cleaned in distilled water for 5 min and air-dried (CD-4800 Digital Ultrasonic Cleaner; Jeken, Dongguan, China). The thicknesses of the specimens were measured using a digital micrometer (Absolute Digimatic Caliper; Mitutoyo, Kawasaki, Japan). All samples were then stored in distilled water for 24 h at 37 °C in an incubator (UM 400; Memmert GmbH, Schwabach, Germany). Surface roughness (Ra) was measured in micrometers (μm) using a profilometer (Mahr GmbH, Göttingen, Germany) before (Ra1) and after the prophylactic paste procedures (Ra2). The difference in surface roughness (ΔRa) was calculated by subtracting Ra1 from Ra2. Baseline color measurements were recorded using Lab* values. Lab* values were obtained using a spectrophotometer (CM-2600d; Konica Minolta Sensing Inc., Tokyo, Japan) against a white (L* = 99.41, a* = −0.07, and b* = −0.20), non-reflective background. Observations were conducted with a 10° observer and illuminant D65.

The sample size determination was based on the findings of a prior study [4]. It was established that a minimum of four specimens per group would provide a statistical power of 0.8, with a significance level of 0.05.

A total of 120 samples, with 30 in each group, were obtained from four different materials. After grouping the samples, polishing paste was initially applied, followed by immersion in a coffee solution. The color selection of the samples was based on the A2 HT shade, commonly used in both the posterior and anterior regions, with blocks corresponding to this color examined in each material after consultation with the manufacturers. Samples for each material were randomly divided into five main groups based on the type of polish to be applied using simple randomization. (Table 2) Polishing paste was applied to five samples from each group (*n* = 5), while five samples served as the control group. Additionally, five samples were allocated for SEM microscope imaging.

According to the determined study categories, the first set of five samples for each CAD/CAM material underwent the application of CLE paste, while the second set received CLJ paste. The third set was treated with DET paste, and the fourth set was subjected to PXT paste. Polishing compounds were applied to the individual surfaces of the materials using a micromotor running at 3000 rpm by the same operator. This process involved a stepwise reduction in grain size, utilizing a rubber brush (Pro-Cup light blue, Kerr, Rastatt, Germany), following the manufacturer’s guidelines. The application time was standardized at 60 s. Both CLE and DET pastes, being single-stage, were applied for 60 s each. In contrast, the three-stage processes of CLJ and PXT pastes were spread over a total of 60 s, divided into three stages, each lasting 20 s. After the applications, any residues were eliminated using steam. Subsequently, the samples underwent drying, and secondary measurements of surface roughness and color coordinates were taken and recorded using a spectrophotometer device.

To assess the impact of PPPs on the surfaces of CAD/CAM materials, additional specimens (five for each material in each group) were specially prepared, coated with gold-palladium, and observed using a field-emission scanning electron microscope (FESEM) device (Zeiss, Gemini 500; Carl Zeiss AG, Oberkochen, Germany). SEM micrographs were taken at ×1000 magnification for visual assessment, using the following imaging parameters: acceleration voltage (EHT) of 5.00 kV, a working distance (WD) that varied (typically ranging from 7.2 mm to 7.9 mm), and secondary electron detection mode (InLens). All micrographs were acquired under identical conditions.

Then, all specimens (*n* = 5) were immersed in a coffee solution within a 37 °C incubator (UM 400; Memmert GmbH, Schwabach, Germany) for 12 days, equivalent to a year of coffee exposure, following the manufacturer’s instructions. After 12 days, the specimens were rinsed with tap water and dried using Selpak tissue paper (Selpak; Eczacıbaşı, Istanbul, Turkey). Subsequently, the third round of spectrophotometric analyses was conducted. Color differences between baseline measurements and after prophylactic paste application (ΔE1) and after immersion in the coffee solution (ΔE2) were computed using the CIELAB (ΔE*ab) and CIEDE2000 (ΔE00) formulas.

$$∆E*ab={\left([{∆L]}^{2}+{\left[∆a\right]}^{2}+{\left[∆b\right]}^{2}\right)}^{1/2}$$

$$∆E00=[(\frac{∆L}{kL}\times SL{)}^{2}+(\frac{∆C}{kC}\times SC{)}^{2}+(\frac{∆H}{kH}\times SH{)}^{2}+RT\times (\frac{∆C}{kC}\times SC{)}^{2}+(\frac{∆H}{kH}\times SH{)}^{2}{]}^{1/2}$$

The parameters kL, kC, and kH in the CIEDE2000 formula were set to 1.35 [13]. The Whiteness Index (WID) was calculated based on CIELAB parameters after PPP application (WID1) and immersion in the coffee solution (WID2) [20]:

WID = 0.511L* − 2.324a* − 1.100b*

### Statistical Analysis

During the analysis of the study outcomes, statistical evaluations were conducted utilizing IBM SPSS Statistics 22. The normality of the data distribution was verified through the Shapiro–Wilks test. A two-way ANOVA test was employed to investigate the collective influence of material type and prophylactic polishing paste (PPP) application on ΔE, ΔWID, and ΔRa. One-way ANOVA was used as a continuation test. Subsequently, post hoc analysis was carried out using the Tukey HDS test. Additionally, Pearson correlation analysis was utilized to explore associations among the variables. The significance level was set at *p* < 0.05.

## 3. Results

**Figure 2:**
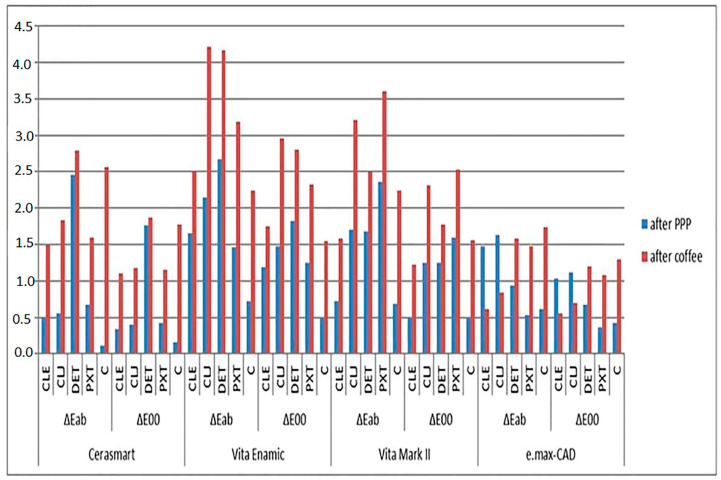
ΔE00 and ΔE*ab values of the tested materials after PPP and immersion in coffee solution.

A two-way ANOVA analysis indicated that both the CIEDE LAB and CIEDE 2000 color systems exhibited significant impacts of material type, polishing system, and their interaction on color change after polishing (ΔE*ab 1; ΔE001) (*p* = 0.001). The alteration in whiteness (ΔWID1) was notably affected by both the material type and polishing system, along with their interaction (*p* = 0.001) (Table 3, Table 4 and Table 5). The specific findings are elucidated in Figure 2 based on the results of the one-way ANOVA test and Tukey HDS tests.

### 3.1. Color Change Across Groups

In all groups, the VE-DET exhibited the highest ΔE*ab1 and ΔE001 values, while the VE-PXT paste group showed the highest ΔWID1. In addition, the CS-C group had the lowest ΔE*ab1, ΔE001 and ΔWID1 values.

For CS specimens, the mean ΔE*ab1, ΔE001, as well as ΔWID1 for the C group, were the lowest and demonstrated a statistically significant difference from the DET group (*p* = 0.007 for ∆E*ab1; *p* = 0.001 for ∆E001; *p* = 0.010 for ∆WID1).

In VE specimens, the mean ΔWID1 values of all paste groups except for the CLE group exhibited higher values than the control group (*p* = 0.001 for CLE vs. DET; *p* = 0.001 for CLE vs. VM).

Concerning VM specimens, all paste groups except CLE displayed higher mean ΔE001 and ΔE*ab 1 values than the C group (*p* = 0.027 for ∆Eab1; *p* = 0.035 for ∆E001). For E.MAX specimens, the mean values of ΔE*ab1 and ΔE001 were observed to be higher than controls for CLE and CLJ groups (*p* = 0.001 for both ∆E*ab1 and ∆E001). However, no differences were found regarding the mean ΔWID1 values for both VM and E.MAX specimens (*p* = 0.288 for VM; *p* = 0.257 for E.MAX).

### 3.2. Prophylaxis Paste Impact on Color Differences

When examining the color differences after prophylaxis paste application, it was found that the ΔE*ab values were statistically significantly lower in CS and VM compared to VE and E.MAX when CLE was used (*p* = 0.001 for CS vs. VE; *p* = 0.003 for VM vs. VE). No significant differences were observed among the other groups (*p* > 0.05). Similarly, when CLJ was used, CS, and VM and E.MAX showed statistically significantly lower ΔE*ab values compared to VE (*p* = 0.001 for CS vs. VE; *p* = 0.001 for VM vs. VE). However, no significant difference was found among the other groups (*p* > 0.05). Following the application of DET paste, E.MAX exhibited lower ΔE*ab values compared to CS (*p* = 0.001) and VE, while no significant difference was observed among the other groups (*p* > 0.05). Moreover, after PXT paste application, E.MAX showed the lowest and VM showed the highest ΔE*ab values, with no significant difference observed between CS and E.MAX (*p* > 0.05).

### 3.3. Whiteness Differences Across Pastes

When evaluating ∆WID1 according to the different pastes, the mean ∆WID1 value for the E.MAX group with the application of PXT paste was significantly lower than that of the VE (*p* = 0.001) and VM (*p* = 0.001) groups (*p* < 0.05). Similarly, the mean ∆WID1 value for the CS group was significantly lower than that of the VM (*p* = 0.001) and VE (*p* = 0.001) groups (*p* < 0.05). No statistically significant differences were observed among the other materials (*p* > 0.05). Additionally, the ∆WID1 values did not show significant differences among materials within the other paste groups (*p* > 0.05).

### 3.4. Clinically Acceptable Thresholds After PPP

Following the PPP treatment, the VE-DET group demonstrated a color alteration exceeding the clinically acceptable threshold according to the CIEDE 2000 system (ΔE00 ≤ 1.8). The whiteness difference for VE-DET and VE-PXT materials was observed to surpass the clinically acceptable level (ΔWID1 ≤ 2.6).

### 3.5. Color and Whiteness Changes After Immersion in Coffee Solution

The two-way ANOVA analysis conducted for the color and whiteness change after immersion in the coffee solution (ΔE002; ΔE*ab2; ΔWID2) demonstrated that both the materials and PPP agents, as well as their interactions, had a substantial influence on the observed results (Table 6, Table 7 and Table 8).

Detailed findings are depicted in Figure 2, based on the outcomes of the one-way ANOVA test and Tukey HDS tests.

Across all groups, the VE CLJ group exhibited the highest values for ΔE*ab 2, ΔE002, and ΔWID2, while the E.MAX-CLE group showed the lowest ΔE*ab 2, ΔE002, and ΔWID2 values.

The mean values of ΔE002, ΔE*ab 2, and ΔWID2 for the control group among the PPP groups were the highest in the CS specimens (*p* = 0.001 for ∆E*ab2; *p* = 0.001 for ∆E002; *p* = 0.001 for ∆WID2). Significant differences were observed in the mean values of ΔE*002 and ΔWID2 between the CLE, CLJ, PXT, and C groups (*p* = 0.001 for ∆E*ab 2 and ∆E002; *p* = 0.002 for ∆WID2). However, the mean value of ΔE*ab 2 was significantly different from the control group for CLE and PXT (*p* = 0.001 for CLE; *p* = 0.002 for PXT).

For the VE specimens, the mean ΔE002, ΔE*ab 2 were higher than control for the PXT, DET, and CLJ groups (*p* = 0.001 for PXT vs. control; *p* = 0.001 for DET vs. control; *p* = 0.001 for CLJ vs. control). The DET and CLJ groups also showed higher ΔE002, ΔE*ab 2 values than CLE and PXT (*p* = 0.001 for DET vs. CLE; *p* = 0.001 for CLJ vs. PXT). No significant differences were found between the other groups (*p* > 0.05). The lowest ΔWID2 was registered for the PXT group and significantly different from all other groups (*p* = 0.001). ΔWID2 for CLJ was higher than from CLE, PXT, and C (*p* = 0.001).

In the realm of VM, no noticeable distinctions were observed in whiteness parameters when compared to the control groups (*p* > 0.05). As for color parameters, the mean ΔE002, ΔE*ab 2 for CLE were lower than for the CLJ and PXT groups (*p* = 0.001 for CLE vs. CLJ and PXT). No differences were observed among the other groups (*p* > 0.05).

For E.MAX specimens, color values for the C and CLJ groups were found to be lower than those observed in the other groups (*p* = 0.001 for C vs. E.MAX; *p* = 0.001 for CLJ vs. E.MAX). No differences were found among the other groups (*p* > 0.05). ΔWID2 for CLE was lower than for DET, PTX, and C (*p* = 0.001). CLJ was lower than DET and C (*p* < 0.05). No differences were found among the other groups (*p* = 0.001).

From the perspective of color changes induced by coffee after polishing, CLE had a noticeable impact on the materials, especially on VE, resulting in significantly higher levels of ΔE002, ΔE*ab 2, and ΔWID2 compared to other materials (*p* = 0.001). Additionally, VM also exhibited higher values of ΔE002, ΔE*ab 2, and ΔWID2 than E.MAX (*p* = 0.001). CS showed higher mean values of ΔE002 and ΔE*ab 2 compared to E.MAX (*p* = 0.001).

Following the application of CLJ, VE and VM exhibited a statistically significant increase in ΔE002 compared to CS and E.MAX (*p* = 0.001), with no notable difference observed between VM and CS, and VE and VM (*p* > 0.05). Similar results were obtained for ΔE*ab 2. For ΔWID2, VE showed statistically significant higher values compared to all other groups (*p* = 0.001). No difference was observed among the other groups (*p* > 0.05).

When DET paste was used, the VE group exhibited significantly higher mean ΔE00 values after exposure to coffee compared to the CS, VM, and E.MAX groups (*p* = 0.001). The mean ΔE00 value of the VM group was lower than that of VE (*p* = 0.001) but significantly higher than that of the E.MAX groups (*p* = 0.001). Furthermore, the mean ΔE00 value of the E.MAX group was significantly lower than those of the CS, VE, and VM groups (*p* > 0.05). Similar results were recorded for ΔE*ab 2, with the only difference being that there was no statistical difference between VM and E.MAX (*p* > 0.05). The comparison of whiteness revealed that the mean ΔWID2 of the VE group was significantly higher than that of the VM (*p*: 0.010) and E.MAX (*p*: 0.019) groups (*p* < 0.05). There was no statistically significant difference among the other materials (*p* > 0.05).

With PXT polishing, VE and VM exhibited significantly greater values of ΔE002, ΔE*ab 2, and ΔWID2 compared to CS and E.MAX (*p* < 0.05), with no significant distinction among the other materials (*p* = 0.001). No significant distinction was noted among other materials (*p* > 0.05).

### 3.6. Clinically Acceptable Thresholds After Coffee

Following the immersion in the coffee solution, the color differences identified in the CS-DET VE-CLJ, VE-DET, VE-PXT, VM-CLJ, and VM-PXT groups surpassed the clinically acceptable thresholds (ΔE*ab ≤ 2.7; ΔE00 ≤ 1.8). Additionally, the mean ΔWID2 for CS-DET, CS-C, VE-C, VE-CLJ, VE-DET, VE-C, all VM groups, and E.MAX-DET, E.MAX-C exceeded the clinically acceptable levels (ΔWID2 ≤ 2.6).

### 3.7. Surface Roughness (Ra Parameter) Analysis

Regarding the Ra parameter, a comprehensive analysis is provided in Table 9. The outcomes of the two-way ANOVA reveal the significant impact of materials, paste application, and their interactions on the surface roughness alterations (ΔRa) following PPP application (Table 10). Across all study groups, after PPP application, the CS-DET group showcased the most substantial change in mean Ra values (*p* < 0.001), whereas the E.MAX-C group displayed the least pronounced alteration (*p* = 0.412). For CS, all the PPP groups statistically significantly changed the surface roughness values compared to control (*p* < 0.001). ΔRa for DET and CJ was higher than those of PXT (*p* < 0.007), CLE (*p* < 0.001), and C (*p* < 0.001). There were no differences between the other groups (*p* > 0.05).

The VE change in mean Ra values was lower than CLJ (*p* < 0.001) and PXT (*p* < 0.001). There were no differences between the other groups (*p* < 0.001).

VM-CLJ (*p* < 0.001) and PXT (*p* < 0.001) created rougher surfaces than the controls (*p* < 0.05). No differences were observed between the other groups (*p* > 0.05).

For E. max, ΔRa for the CLE (*p* < 0.001) and C groups (*p* < 0.001) were lower than the CLJ-DET (*p* < 0.001) and CLJ-PXT groups (*p* < 0.001). There were no differences between the other groups (*p* > 0.05).

### 3.8. SEM Microphotographs and Correlations

**Figure 3:**
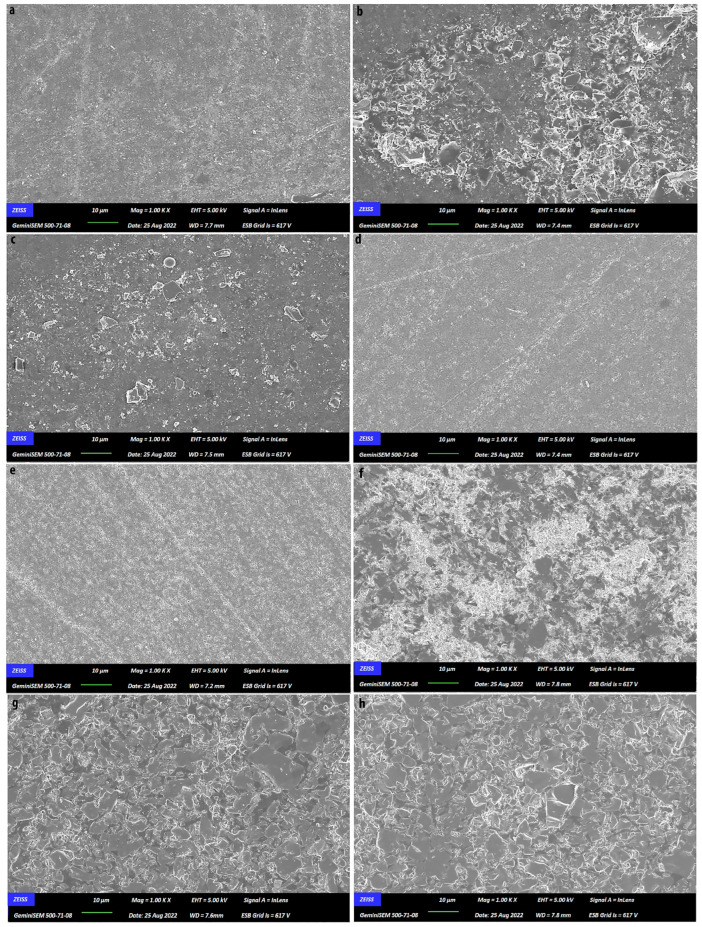

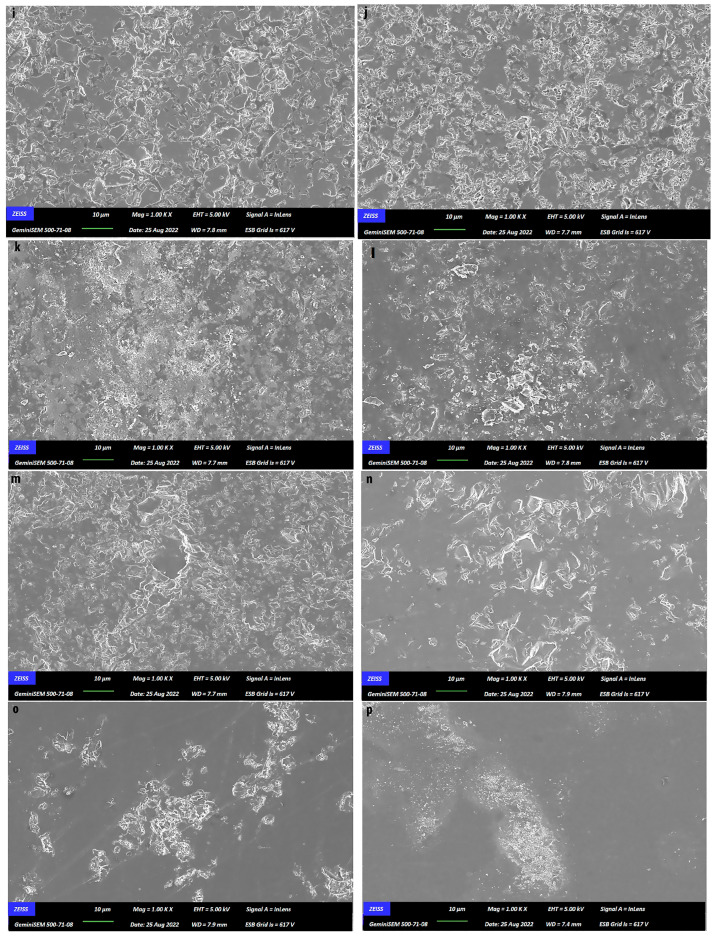

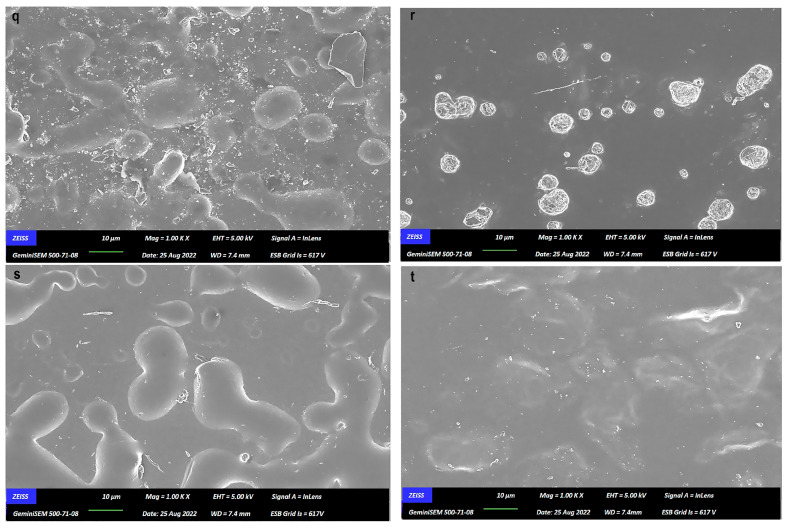
Scanning electron microscopy analysis (×1000 magnification). (**a**) CS-CLJ; (**b**) CS-DET; (**c**) CS-CLE; (**d**) CS-PXT; (**e**) CS-C; (**f**) VE-CLJ; (**g**) VE-DET; (**h**) VE-CLE; (**i**) VE-PXT; (**j**) VE-C; (**k**) VM-CLJ; (**l**) VM-DET; (**m**) VM-CLE; (**n**) VM-PXT; (**o**) VM-C; (**p**) E.MAX-CLJ; (**q**) E.MAX-DET; (**r**) E.MAX-CLE; (**s**) E.MAX-PXT; (**t**) E.MAX-C.

In SEM microphotographs (Figure 3), the distinctions in both inter-material and intra-material impacts of the pastes are readily discernible compared to groups C.

**Figure 4:**
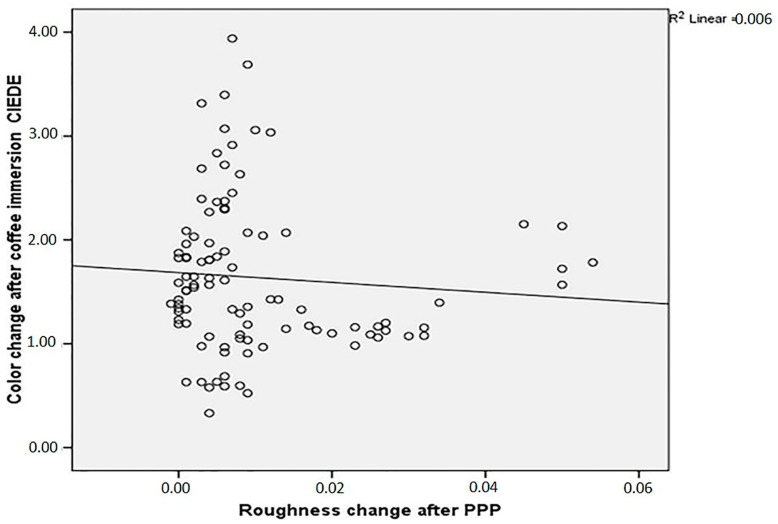
Correlation between change in surface roughness after PPP (ΔRa) and color change of specimens after immersion in coffee solution (ΔE002).

**Figure 5:**
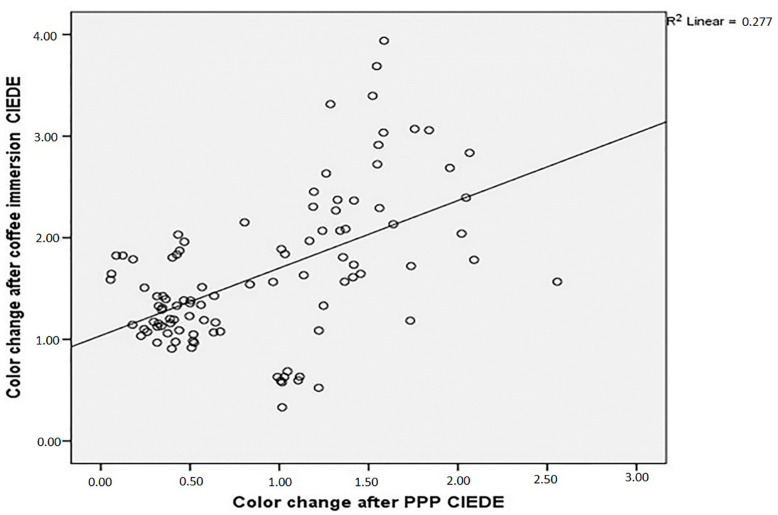
Correlation between color change after PPP (ΔE001) and color change of specimens after immersion in coffee solution (ΔE002).

The analyzes of the data showed that there was no correlation between roughness change after PPP and ΔE00 (r: −0.80; *p* > 0.05) (Figure 4). However, ΔE001 and ΔE002 statistically significantly have a moderate correlation (r: 0.52; *p* < 0.05) (Figure 5).

### 3.9. Effect of Single vs. Multi-Stage Pastes

The results of the two-way ANOVA also indicated that whether pastes were single or multi-stage has a significant effect on roughness after PPP (F: 9.047; *p*< 0.05).

## 4. Discussion

In the present study, the effect of prophylaxis pastes (PPPs) on the color, surface roughness, and stainability of CAD/CAM resin ceramics was investigated. The initial null hypothesis that “Prophylactic pastes have no effect on the stainability of CAD/CAM resin restorative materials” was rejected. The findings revealed that the stain resistance of some tested CAD/CAM restorative materials was affected by PPP application.

Previous research has emphasized the influence of factors such as crystalline structure, polymeric matrix, filler size, and form on material surface quality and discoloration rates [21,22]. Additionally, materials containing more resilient components tend to undergo uniform discoloration, leading to increased color stability [23]. IPS E.MAX CAD is characterized by a high content of crystals embedded in a glassy matrix, with crystal diameters ranging from 0.2 to 1 μm. A higher crystalline ratio generally enhances mechanical properties [4,24,25]. VM is made up of feldspathic crystalline particles, usually between 1 and 10 μm in size, incorporated within a glassy matrix [4]. VE typically features a sintered porous ceramic network reinforced by a polymer network, with mechanical properties falling between porcelain and resin composites [26]. On the other hand, CS is a composite resin containing ultrafine glass particles within a highly cross-linked resin matrix [4]. The variations among these materials could account for their discrepant stain resistance.

In this investigation, all the control groups exhibited perceptible color changes after exposure to coffee, which remained within clinically acceptable limits. Following coffee immersion, E.MAX specimens showed less color alteration compared to the other groups, while the most pronounced change was observed in the CS group, consistent with the structures of the tested materials. Similar results have been found in previous research, especially related to the stain susceptibility of hybrid ceramics. Acar et al. [27] showed that thermal cycling in coffee caused a perceptible color change in VE (ΔE00 > 1.28), while IPS E.MAX CAD had barely noticeable changes (ΔE00 ≤ 1.28). Barutcugil et al. [28] also reported color changes above clinically acceptable limits (ΔE00 > 2.25) in CS and VE after one month of exposure to staining solutions. According to Lawson’s report [29], VE exhibited lower stain resistance compared to E.MAX CAD; however, both materials remained within the limits of clinically acceptable color change (ΔE00 ≤ 2.23). Abu-Obaid et al. [19] found that, after glazing, VE showed the highest discoloration rate, followed by VM (ΔEab < 3.3). Kanat-Ertürk et al. [30] reported that E.MAX had lower color changes (ΔEab = 1.65) compared to zirconia-reinforced lithium silicate ceramic following 2-month storage in beverages, representing the role of surface finishing. Adawi et al. [31] found that VE showed higher discoloration after immersion in coffee (ΔE00 = 2.90) compared to VM (ΔE00 = 2.79), revealing that stainability is material-depending.

The surface roughness after polishing is influenced by both the composition of the material and the characteristics of the prophylaxis paste used [7,32]. Previous research has demonstrated that abrasive particles in prophylactic pastes can alter surface roughness by eliminating microparticles from various restorative materials, including composites [33], indirect composites [9], feldspathic porcelain [15], CAD/CAM glass ceramics, lithium disilicate ceramics [17], and composite resin blocks [31].

In the present study, prophylaxis pastes affected the surface roughness of the tested materials to different degrees. However, none of the materials showed Ra values exceeding the 0.2 µm threshold linked to increased microbial adhesion and a higher risk of caries or gingivitis [4,10,11].

In contrast to the findings of Liebermann et al. [6], this study showed that single-stage pastes (e.g., Detartrine) caused significantly higher surface roughness than multi-stage pastes (e.g., Proxyt). This difference may be due to the study design, as the current study tested four different materials, while the other study tested only one. These results suggest that not only the grain size but also their shape and hardness, the viscosity of the paste, the time of application, and the pressure used can affect surface roughness [9].

Different surface roughnesses influence material stainability [34]. In this study, CLE and PTX reduced staining in CS, while CLE and CLJ did the same for E.MAX. However, staining susceptibility increased for VE-CJ and VE-DET. Despite expecting a link between surface roughness and stainability, the correlation analysis found no relationship, contrary to previous studies [4,34]. Therefore, the second null hypothesis, stating no correlation between surface roughness change and discoloration, was accepted.

In this regard, the difference in discoloration observed among the tested materials after coffee exposure may stem from the effects of colorants [33] or substances such as alcohol present in the pastes, exerting an influence on the color and whiteness of the material. This statement is supported by the findings of this study, indicating a moderate positive correlation between changes in color and whiteness following prophylaxis and changes in color and whiteness following exposure to coffee.

The CIEDE2000 formula has been observed to offer a more accurate alignment with the visually determined acceptability and detectability of color disparities in dental ceramics [35]. However, dental research traditionally relies predominantly on the CIELAB color difference formula for reporting results. Consequently, in this investigation, color distinctions were evaluated using both the CIEDE2000 and CIELAB color difference formulas. Consistent with prior research [36], an examination of the results from this study unveiled a notable correlation between ΔE00 and ΔE*ab.

In addition to assessing color, it is advisable to accurately measure whiteness for both natural teeth and restorative materials in research and clinical settings. In this study, we utilized the Whitening Index (WID) for evaluating whiteness, a dental-specific index known for its superior performance compared to previous methods [13,14].

In the present investigation, specimens were fabricated using a low-speed cutting instrument, a method employed in numerous prior studies. However, the absence of CAD-CAM systems in specimen preparation constitutes a limitation in this study.

Polishing each surface for a minute with a specific paste differs from real-world clinical application, where shorter polishing times and different pressures are common, potentially affecting the results.

The fact that in this study used only Ra, the most widely preferred parameter, to measure surface roughness is another limitation of this research. Using more parameters would provide a better understanding of the surface properties.

Coffee was chosen as our testing solution for stainability assessment due to its extensive use in daily life, high chromogenic content, and acidic pH [34,37]. However, previous research has shown that variations in discoloration on CAD/CAM materials may be due to the type of solution and the exposure time [34].

Further in vitro and clinical studies with different coloring solutions and variety of CAD/CAM materials are necessary to fully corroborate the findings of our research. Additionally, prolonged immersion staining might not entirely reflect real-world clinical scenarios; in the oral cavity, exposure to staining agents from foods or beverages occurs occasionally, and the stains can be diluted with saliva or other fluids.

In this experimentation, both surfaces of the materials were impacted during immersion in the coffee solution. However, considering that restorations are cemented onto teeth within the oral environment, typically only one surface of the materials is exposed. This aspect has also influenced the study outcomes.

## 5. Conclusions

Within the limitations of this study, the following conclusions can be drawn:

1. CAD/CAM restorative materials show a material-specific response to the influence of prophylactic polishing pastes on color and surface roughness.
2. There was no discernible relationship found between the surface roughness resulting from the application of prophylactic polishing pastes and the discoloration of CAD/CAM materials.
3. According to both approaches for color measurement, a moderate positive correlation was noted between ΔE1 and ΔE2.

## Figures and Tables

**Table 1:** Composition of the tested materials.

Material	Type	Chemical Content (Weight %)	Color	Particle Size	Manufacturer
Cerasmart 270	Nanoceramic	Bis-MEPP, UDMA, DMA Silicon dioxide (SiO_2_), barium glass	A2 HT (High translucent)		GC Corp., Tokyo, Japan
IPS Emax CAD	Lithium disilicate based glass-matrix ceramic	58–80% SiO_2_, 11–19% Li_2_O, 0–13% K_2_O, 0–8% ZrO_2_, 0–5% Al_2_O_3_	A2-HT		Ivoclar Vivadent
Vita Enamic	Hybrid ceramic	86% inorganic (58–63% SiO_2_, 20–23% Al_2_O_3_, 9–11% Na_2_O, 4–6% K_2_O, 0.1% ZrO_2_), 14% organic	2M2-C-HT		VITA Zahnfabrik
VITABLOCS Mark II for Cerec	Feldspathic glass-matrix ceramic	56–64% SiO_2_, 20–23% Al_2_O_3_,6–9% Na_2_O, 6–8% K_2_O	2M2-C		VITA Zahnfabrik, Bad Säckingen, Germany
Cleanic	Prophylactic polishing paste (PPP)	Titanium dioxide, glycerine, sodium fluoride < 0.25%, ethanol < 1%		RDA 21	Kerr, Rastatt, Germany
CleanJoy	Prophylactic polishing paste (PPP)	Surfactant < 2.5%, peppermint flavor < 2.5%, natriumfluoride < 2.5%		Coarse RDA 195; Medium RDA 127; Fine RDA 16	Voco, Cuxhaven, Germany
Detartrine	Prophylactic polishing paste (PPP)	Quartz 25–50%, glycerine 10–25%, ethanol < 2.5%, zircon in silicate		RDA 150	Septodont, Saint-Maur-des-Fosses, France
Proxyt	Prophylactic polishing paste (PPP)	Water, glycerine 41.0; sorbite, xylit 21.0; inorganic fillers 35.0; excipients 1.2; natriumfluoride 0.12		Coarse RDA 83; Medium RDA 36; Fine RDA 7	Ivoclar Vivadent, Schaan, Liechtenstein

**Table 2:** Letters-code designations of the groups.

Material	Prophylactic Polishing Paste (PPP)	Code
VITABLOCS Mark II for Cerec	Cleanic	VM-CLE
VITABLOCS Mark II for Cerec	CleanJoy	VM-CLJ
VITABLOCS Mark II for Cerec	Detartrine	VM-DET
VITABLOCS Mark II for Cerec	Proxyt	VM-PXT
VITABLOCS Mark II for Cerec	Control	VM-C
IPS Emax CAD	Cleanic	E.MAX-CLE
IPS Emax CAD	CleanJoy	E.MAX-CLJ
IPS Emax CAD	Detartrine	E.MAX-DET
IPS Emax CAD	Proxyt	E.MAX-PXT
IPS Emax CAD	Control	E.MAX-C
Vita Enamic	Cleanic	VE-CLE
Vita Enamic	CleanJoy	VE-CLJ
Vita Enamic	Detartrine	VE-DET
Vita Enamic	Proxyt	VE- PXT
Vita Enamic	Control	VE-C
Cerasmart 270	Cleanic	CS-CLE
Cerasmart 270	CleanJoy	CS-CLJ
Cerasmart 270	Detartrine	CS-DET
Cerasmart 270	Proxyt	CS-PXT
Cerasmart 270	Control	CS-C

**Table 3:** Assessment of the impact of material and polishing application method on ∆E*ab after prophylaxis.

∆E*ab1	Type III Sum of Squares	df	Mean Square	F	*p*
Material	11,571	3	3857	24,944	0.001 *
Polishing Method	21,624	4	5406	34,963	0.001 *
Material * Polishing	21,345	12	1779	11,504	0.001 *

Two-way ANOVA test, * *p* < 0.05.

**Table 4:** Assessment of the impact of material and polishing application method on ∆E00 after prophylaxis.

∆E001	Type III Sum of Squares	df	Mean Square	F	*p*
Material	12,063	3	402	53,967	0.001 *
Polishing Method	4659	4	1165	15,632	0.001 *
Material * Polishing	11,203	12	0.934	12,530	0.001 *

Two-way ANOVA test, * *p* < 0.05.

**Table 5:** Assessment of the impact of material and polishing application technique on ∆WID1 following prophylaxis.

∆WID1	Type III Sum of Squares	df	Mean Square	F	*p*
Material	20,701	3	6900	11,744	0.001 *
Polishing Method	35,265	4	8816	15,005	0.001 *
Material * Polishing	26,110	12	2176	3703	0.001 *

Two-way ANOVA test, * *p* < 0.05.

**Table 6:** Assessment of the impact of material and polishing application method on ∆E*ab after coffee.

∆E*ab2	Type III Sum of Squares	df	Mean Square	F	*p*
Material	55,072	3	18,357	60,091	0.001 *
Polishing Method	17,313	4	4328	14,168	0.001 *
Material * Polishing	23,966	12	1997	6538	0.001 *

Two-way ANOVA test, * *p* < 0.05.

**Table 7:** Assessment of the impact of material and polishing application method on ΔE00 following exposure to coffee.

∆E002	Type III Sum of Squares	df	Mean Square	F	*p*
Material	10,939	3	3646	38,540	0.001 *
Polishing Method	4738	4	1185	12,521	0.001 *
Material * Polishing	10,670	12	0.889	9398	0.001 *

Two-way ANOVA test, * *p* < 0.05.

**Table 8:** Assessment of the impact of material and polishing application method on ∆WID after coffee exposure.

∆WID2	Type III Sum of Squares	df	Mean Square	F	*p*
Material	65,777	3	21,926	14,926	0.001 *
Polishing Method	29,317	4	732	4989	0.001 *
Material * Polishing	76,726	12	639	4353	0.001 *

Two-way ANOVA test, * *p* < 0.05.

**Table 9:** Roughness change after prophylaxis.

	∆Ra					
	Cleanic	Cleanjoy	Detartrine	Proxyt	Control	*p*
Material	Mean ± S D	Mean ± SD	Mean ± SD	Mean ± SD	Mean ± SD	
Cerasmart	0.021 ± 0.004	0.031 ± 0.002	0.049 ± 0.004	0.023 ± 0.003	0.001 ± 0.001	0.001 *
VitaEnamic	0.003 ± 0.002	0.006 ± 0.002	0.005 ± 0.002	0.006 ± 0.002	0 ± 0.001	0.001 *
Vita Mark 2	0.005 ± 0.002	0.008 ± 0.005	0.006 ± 0.001	0.009 ± 0.003	0.001 ± 0.001	0.002 *
E.Max	0.003 ± 0.002	0.007 ± 0.001	0.010 ± 0.003	0.010 ± 0.002	0 ± 0	0.001 *
*p*	0.001 *	0.001 *	0.001 *	0.001 *	0.412	

One-way ANOVA and Tukey test, * *p* < 0.05.

**Table 10:** Assessment of the impact of material and polishing application method on ΔRa.

∆Ra	Type III Sum of Squares	df	Mean Square	F	*p*
Material	0.007	3	0.002	415,948	0.001 *
Polishing Method	0.004	4	0.001	146,690	0.001 *
Material * Polishing	0.003	12	0	48,215	0.001 *

Two-way ANOVA test, * *p* < 0.05.

## Data Availability

The original contributions presented in this study are included in the article. Further inquiries can be directed to the corresponding author.

## References

[1] Bona A.D., Pecho O.E., Ghinea R., Cardona J.C., Pérez M.M. (2015). Colour parameters and shade correspondence of CAD-CAM ceramic systems. J. Dent..

[2] Colombo M., Poggio C., Lasagna A., Chiesa M., Scribante A. (2019). Vickers micro-hardness of new restorative CAD/CAM dental materials: Evaluation and comparison after exposure to acidic drink. Materials.

[3] Alessandretti R., Borba M., Benetti P., Corazza P.H., Ribeiro R., Bona A.D. (2017). Reliability and mode of failure of bonded monolithic and multilayer ceramics. Dent. Mater..

[4] Tinastepe N., Malkondu O., Iscan I., Kazazoglu E. (2021). Effect of home and over the contour bleaching on stainability of CAD/CAM esthetic restorative materials. J. Esthet. Restor. Dent..

[5] Wang F., Takahashi H., Iwasaki N. (2013). Translucency of dental ceramics with different thicknesses. J. Prosthet. Dent..

[6] Liebermann A., Spintzyk S., Reymus M., Schweizer E., Stawarczyk B. (2019). Nine prophylactic polishing pastes: Impact on discoloration, gloss, and surface properties of a CAD/CAM resin composite. Clin. Oral Investig..

[7] Neme A.L., Frazier K.B., Roeder L.B., Debner T.L. (2002). Effect of prophylactic polishing protocols on the surface roughness of esthetic restorative materials. Oper. Dent..

[8] Patil S.S., Rakhewar P.S., Limaye P.S., Chaudhari N.P. (2015). A comparative evaluation of plaque-removing efficacy of air polishing and rubber-cup, bristle brush with paste polishing on oral hygiene status: A clinical study. J. Int. Soc. Prev. Community Dent..

[9] Can Say E., Yurdagüven H., Malkondu Ö., Ünlü N., Soyman M., Kazazoğlu E. (2014). The effect of prophylactic polishing pastes on surface roughness of indirect restorative materials. Sci. World J..

[10] Lutz F., Sener B., Imfeld T., Barbakow F., Schüpbach P. (1993). Self-adjusting abrasiveness: A new technology for prophylaxis pastes. Quintessence Int..

[11] Setcos J.C., Tarim B., Suzuki S. (1999). Surface finish produced on resin composites by new polishing systems. Quintessence Int..

[12] Pecho E.O., Ghinea R., Alessandretti R., Pérez M.M., Bona A.D. (2016). Visual and instrumental shade matching using CIELAB and CIEDE2000 color difference formulas. Dent. Mater..

[13] Pérez M.M., Ghinea R., Rivas M.J., Yebra A., Ionescu A.M., Paravina R.D., Herrera L.J. (2016). Development of a customized whiteness index for dentistry based on CIELAB color space. Dent. Mater..

[14] Paravina R.D., Pérez M.M., Ghinea R. (2019). Acceptability and perceptibility thresholds in dentistry: A comprehensive review of clinical and research applications. J. Esthet. Restor. Dent..

[15] Yurdaguven H., Aykor A., Ozel E., Soyman M. (2012). Influence of a prophylaxis paste on surface roughness of different composites, porcelain, enamel and dentin surfaces. Eur. J. Dent..

[16] Fratolin M.M., Bianco V.C., Santos M.J., Rizkalla A.S., Santos G.C. (2014). The effect of prophylactic powders on the surface roughness of enamel. Compend. Contin. Educ. Dent..

[17] Monaco C., Arena A., Ozcan M. (2014). Effect of prophylactic polishing pastes on roughness and translucency of lithium disilicate ceramic. Int. J. Periodontics Restor. Dent..

[18] Monaco C., Arena A., Scheda L., Fiore D.A., Zucchelli G. (2020). In vitro 2D and 3D roughness and spectrophotometric and gloss analyses of ceramic materials after polishing with different prophylactic pastes. J. Prosthet. Dent..

[19] Abu-Obaid A., AlMawash A., Alyabis N., Alzaaqi N. (2020). An in vitro evaluation of the effect of polishing on the stainability of different CAD/CAM ceramic materials. Saudi Dent. J..

[20] CIE (2004). Colorimetry. Report No.: CIE Pub. No. 15.

[21] Koller M., Arnetzl G.V., Holly L., Arnetzl G. (2012). Lava ultimate resin nano ceramic for CAD/CAM: Customization case study. Int. J. Comput. Dent..

[22] Koizumi H., Saiki O., Nogawa H., Hiraba H., Okazaki T., Matsumura H. (2015). Surface roughness and gloss of current CAD/CAM resin composites before and after toothbrush abrasion. Dent. Mater. J..

[23] Kilinc H., Turgut S. (2017). Optical behaviors of esthetic CAD-CAM restorations after different surface finishing and polishing procedures and UV aging: An in vitro study. J. Prosthet. Dent..

[24] Turker S.B., Biskin T. (2002). The effect of bleaching agents on the microhardness of dental aesthetic restorative materials. J. Oral Rehabil..

[25] Turker S.B., Biskin T. (2003). Effect of three bleaching agents on the surface properties of three different esthetic restorative materials. J. Prosthet. Dent..

[26] Bona A.D., Corazza P.H., Zhang Y. (2014). Characterization of a polymer-infiltrated ceramic-network material. Dent. Mater..

[27] Acar O., Yılmaz B., Altıntas S.H., Chandrasekaran I., Johnston W.M. (2016). Color stainability of CAD/CAM and nanocomposite resin materials. J. Prosthet. Dent..

[28] Barutçugil B., Bilgili D., Barutcigil K., Dündar A., Büyükkaplan U.S., Yılmaz B. (2019). Discoloration and translucency changes of CAD-CAM materials after exposure to beverages. J. Prosthet. Dent..

[29] Lawson N.C., Burgess J.O. (2021). Gloss and stain resistance of ceramic-polymer CAD/CAM restorative blocks. J. Esthet. Restor. Dent..

[30] Kanat-Ertürk B. (2020). Color Stability of CAD/CAM Ceramics Prepared with Different Surface Finishing Procedures. J. Prosthodont..

[31] Adawi H., Mialeem M., Ahmari N., Shariff M., Qahhar M., Muharraq S., Alghazali N. (2021). Assessment of Color Stainability of Computer-Aided Design and Computer-Aided Manufacturing (CAD/CAM) Ceramic Materials After Hot and Cold Coffee Immersion at Different Time Intervals. Med. Sci. Monit..

[32] Covey D.A., Barnes C., Watanabe H., Johnson W.W. (2011). Effects of pastefree prophylaxis polishing cup and various prophylaxis polishing pastes on tooth enamel and restorative materials. Gen. Dent..

[33] Kuzu T.E., Karatas O. (2023). The effect of prophylaxis paste and air-powder polishing on color stability and surface roughness of different composite resins. J. Oral Res. Rev..

[34] Kursoglu P., Motro P.F., Kazazoglu E. (2014). Correlation of surface texture with the stainability of ceramics. J. Prosthet. Dent..

[35] Ghinea R., Pérez M.M., Herrera L.J., Rivas M.J., Yebra A., Paravina R.D. (2010). Color difference thresholds in dental ceramics. J. Dent..

[36] Lee Y.K. (2005). Comparison of CIELAB DeltaE* and CIEDE2000 color-differences after polymerization and thermocycling of resin composites. Dent. Mater..

[37] Ertaş M., Yıldız E., Ünlü E., Aydın S. (2006). The effect of coffee on the color stability of dental restorative materials. J. Dent. Mater..

